# Visual Attention Modulates Phenomenal Consciousness: Evidence From a Change Detection Study

**DOI:** 10.3389/fpsyg.2019.02150

**Published:** 2019-09-20

**Authors:** Luca Simione, Enrico Di Pace, Salvatore G. Chiarella, Antonino Raffone

**Affiliations:** ^1^Department of Psychology, Sapienza University of Rome, Rome, Italy; ^2^Institute of Cognitive Sciences and Technologies (ISTC), CNR, Rome, Italy; ^3^ECONA, Interuniversity Center, Rome, Italy; ^4^School of Buddhist Studies, Philosophy, and Comparative Religions, Nalanda University, Rajgir, India

**Keywords:** attention, consciousness, working memory, iconic memory, change detection

## Abstract

The distinction between phenomenal and access consciousness has been influential in the field of consciousness studies. Both Block and Lamme proposed that access consciousness, or narrow cognitive accessibility, is related to a limited capacity working memory, and that phenomenal consciousness, or broad cognitive accessibility, is related to iconic memory or, more recently, to a fragile (intermediate) short-term memory store with a larger capacity than working memory. They have also highlighted the preattentive nature of phenomenal consciousness and of the related iconic and fragile visual short-term stores, thus selectively linking attention with access consciousness, in line with Baars and Dehaene, among others. However, a range of electrophysiological and neurophysiological studies suggest that visual attention can affect early responses of neurons in visual cortex, before conscious access. Furthermore, some theories and neurocomputational models suggest earlier attentional biases related to phenomenal consciousness. To solve this controversy, and to shed light on the relationships of attention with iconic memory and subsequent stages of visual maintenance, we conducted an experiment with a novel procedure of change detection based on delayed cueing of the target for report with high- and low-priority objects marked by color. In line with our hypothesis, the results show an attentional bias toward high-priority objects in the memory array with the longer (600 and 1,200 ms) cueing delays associated with a fragile (intermediate) visual short-term memory, but not with the shorter cueing delays (16.6 and 200 ms) associated with iconic memory. These findings therefore suggest two stages of phenomenal consciousness before access consciousness: a first preattentive stage related to iconic memory and a second stage related to fragile visual short-term memory intermediate between iconic and visual working memory, which is modulated by visual attention in a time-dependent manner. Finally, our results suggest the dissociation between a mid-level visual attention modulating phenomenal consciousness and a central attention directing access consciousness.

## Introduction

### Phenomenal and Access Consciousness

Ned Block proposed the influential distinction between phenomenal and access consciousness (Block, 1995, 2005). *Phenomenal consciousness* refers to qualia, i.e., first-person experience contents, as for example in the subjective perceptual experiences of red and green (see also Huxley, 1874; Nagel, 1974; Jackson, 1982). *Access consciousness* represents information which is made available to the neurocognitive systems (“consuming systems”) for working memory, reasoning, planning, decision-making and voluntary control of attention, in association with a *global workspace* in the brain (see also Baars, 1988, 1997; Dehaene et al., 1998).

Block (2007) further suggested that phenomenal consciousness is linked with the notion of *broad cognitive accessibility*, an intermediate level of representation between the unconscious level and access consciousness, with the latter related to *narrow cognitive accessibility*. Thus, according to Block, “the capacity of phenomenology is greater than that of the workspace – so it is narrow accessibility that is at issue” (Block, 2007, p. 492). These three sets of information are related to the taxonomy proposed by Dehaene and Naccache (2001, see also Dehaene et al., 2006), in which some information encoded in the nervous system is inaccessible to a conscious level (set I1), other information is potentially accessible in the global workspace for conscious access, as it can be consciously amplified if it is attended to (set I2), with only one selected content (object) of the latter being at any one time accessed in the workspace for conscious access (set I3). However, unlike Block, Dehaene and Naccache regard the intermediate set I2 in terms of preconscious representations rather than phenomenally conscious contents with broad cognitive accessibility.

A characterization of set I2 in visual perception has been put forth by Lamme (2007): “Since there is little disagreement about the absence of conscious experience in I1, or about its presence in I3, the question becomes whether I2 is more like I1 (i.e., unconscious) or like I3 (conscious)” (p. 512). According to Lamme (2007), neuroscientific findings suggest that set I2 is more closely linked with set I3 than set I1, due to recurrent (or re-entrant, with reciprocal signaling between groups of neurons or brain areas) neural processing in both I2 and I3, versus a non-recurrent or feedforward (unidirectional) processing in I1. Thus, set I2 can be associated to phenomenal consciousness (Lamme, 2003; Block, 2007), characterized by a graded rather than an all-or-none activation as in the global workspace for conscious access (Dehaene et al., 2003). This view of Lamme has been supported by our previous neurocomputational modeling studies showing plausible neural mechanisms for recurrent interactions not only for access consciousness but also for phenomenal consciousness (Raffone and Pantani, 2010; Simione et al., 2012).

Lamme (2003, 2006) and Block (2007, 2011) have linked access consciousness or narrow cognitive accessibility with working memory, and phenomenal consciousness or broad cognitive accessibility with iconic memory. The distinction between iconic memory as preattentive and with a large storage capacity (Sperling, 1960; Averbach and Coriell, 1961) and working memory that requires attention and with a limited storage capacity (Miller, 1956; Luck and Vogel, 1997; Cowan, 2001; Raffone and Wolters, 2001) is closely related to the overflow argument of Block, as well as to his theoretical distinction between phenomenal and access consciousness. According to Block (2011, p. 567) “the overflow argument appeals to visual iconic memory to argue that a conscious perceptual system that has ‘rich’ contents ‘overflows’ – that is, has a higher capacity than – the ‘sparse’ system that cognitively accesses perception.” Thus, iconic memory is taken as instance of phenomenal consciousness, which is characterized by rich contents and overflows working memory and access consciousness, the spare system which demands attention (Lamme, 2004; Koch and Tsuchiya, 2007; Aru and Bachmann, 2013).

### Stages of Visual Processing and the Role of Attention

Although the role of attention for the transfer of visual information to working memory is established (e.g., Atkinson and Shiffrin, 1968; Lamme, 2003; Vogel and Machizawa, 2004; Simione et al., 2012; Raffone et al., 2014), the independence of iconic memory from attention is controversial. Indeed, some authors share the view that iconic representation is attention-free and overflows attentional access (Block, 1995, 2011, 2014; Lamme, 2004; Koch and Tsuchiya, 2007; Aru and Bachmann, 2013; Bronfman et al., 2014), while others regard phenomenal perception as not attention-free (Mack and Rock, 1998; Cohen et al., 2012; Persuh et al., 2012; Mack et al., 2015).

Iconic memory, which is associated to phenomenal consciousness, and visual short-term (working) memory, which is related to access consciousness, are both key components of the classic modal model of memory (Atkinson and Shiffrin, 1968). This model includes a sensory memory (e.g., iconic memory), which maintains information for a very few hundred milliseconds with a very large capacity, and a short-term memory (later reconceptualized as working memory; Baddeley and Hitch, 1974), with a severely limited capacity but with a more robust and longer maintenance. With relevance for a neurocognitive characterization of phenomenal and access consciousness, recent findings suggest an intermediate stage between iconic memory and visual short-term (working) memory, in terms of a fragile visual short-term memory (Sligte et al., 2008), which has been linked with phenomenal consciousness (Block, 2011). Evidence suggests that this intermediate store is characterized by a capacity more limited than iconic memory, but with an almost twice capacity than visual short-term (working) memory (Landman et al., 2003; Sligte et al., 2008, 2009, 2010, 2011; Vandenbroucke et al., 2011, 2015).

In a behavioral study, Vandenbroucke et al. (2011) dissociated the stage of fragile visual short-term memory, related to phenomenal consciousness, from visual working memory, related to access consciousness, by means of three experiments in which they manipulated attentional processing. These manipulations diverted the attention during performance of a change detection task that measured the storage capacity of both the fragile visual short-term memory and visual working memory. In this study, attention was manipulated in three ways: in the first experiment by temporal uncertainty of the presentation of the memory array (Coull et al., 2000); in the second and third experiments, they used a dual task design by coupling the cue change detection task with a rapid serial visual presentation (RSVP) and an attentional blink procedure (Raymond et al., 1992). In all the three experiments, the results showed a significant decrease of working memory capacity with a diverted attention, while the capacity of the fragile visual short-term memory was only slightly affected by the attentional manipulations. This evidence appears to suggest that unlike visual working memory related to conscious access, the memory trace in fragile short-term memory related to phenomenal consciousness does not depend on attention.

A number of neuroscientific findings in experiments involving humans (through event-related potentials) and animals (through single cell recordings), however, suggest that visual attention operates since early stages in visual processing (e.g., Luck et al., 2000; Bisley, 2011; Raffone et al., 2014), thus before the stage of conscious access. In fact, this stage should take place after about 300 ms from stimulus onset through a global workspace neurodynamics involving prefronto-parietal areas (Dehaene et al., 2006; Gaillard et al., 2009). Considering such findings, thus, neural responses in the visual system associated with set I2 for broad cognitive accessibility are predicted to depend on attention rather than being attention-free.

The Theory of Attention and Consciousness (TAC) proposed by Raffone et al. (2014) has suggested multiple stages between early preattentive visual perception and conscious access, through top-down attentional modulation that biases visual responses, a serial guided attentional filtering process (Wolfe, 1994), attentional selection of targets and their intermediate buffering before consolidation and encoding in visual working memory. TAC leads to the prediction of an early iconic memory component which is not biased by top-down attention and of a later fragile visual short-term memory, a stage of visual processing in which top-down attention is hypothesized to bias visual representations before the stages of conscious access and encoding in visual working memory. Such evidence would suggest two components of phenomenal consciousness: an earlier preattentive component related to early iconic memory and a later attention-modulated component related to an intermediate buffer or fragile visual short-term memory.

### Objectives and Hypotheses of the Study

In order to test the prediction derived from TAC and earlier neurocomputational models (Raffone and Pantani, 2010; Simione et al., 2012) and thus contribute to an increased understanding of the relationships between visual attention and phenomenal consciousness, we designed a behavioral experiment based on the revised change detection procedure of Landman et al. (2003) on the basic visual working memory task proposed by Luck and Vogel (1997). A key innovation of our task with memory and probe arrays containing eight oriented items (see also Landman et al., 2003), was to manipulate the priority of the items: half of which with a color marking their higher probability to be selected for cueing and another half with another color marking their lower probability to be selected for cueing. Indeed, feature-based selection (e.g., by color) of a subset of objects in the memory array has been shown to influence storage in visual working memory (Bundesen, 1990; Vogel et al., 2005). Here, we use color for the modulation of object priority rather than for filtering of response-irrelevant distracters. We also manipulated the cue onset, 16.6, 200, 600, or 1,200 ms after the offset of the memory array, to probe the target item.

We thus hypothesized an attentional bias for the longer cueing intervals (600 and 1,200 ms after offset of the memory array), plausibly related to a fragile (intermediate) visual working memory store, but not for the shorter cueing intervals (16.6 and 200 ms after offset of the memory array), plausibly related to a preattentive iconic memory (e.g., Haber and Standing, 1969; Long, 1980). In line with Block and Lamme, we assumed the implication of phenomenal consciousness in association with iconic memory and fragile visual short-term memory. Furthermore, we hypothesized the involvement of two stages of phenomenal consciousness related to such two stages of visual processing, which were differentially modulated by visual attention. Access consciousness was assumed to be implicated after cueing of the target object location, as related to encoding in visual working memory, and at the subsequent retrieval from visual working memory for the change detection report. The use of two colors was moreover likely to prevent perceptual grouping processes based on proximity and similarity, which could have biased the storage capacity estimates in the different conditions.

## Materials and Methods

### Participants

To determine the number of participants for this study, we conducted a G-Power computation under the assumptions to find a moderate to large effect size (>0.6) for the main effects and a medium effect size (0.3–0.4) for the interaction effect, with a power *β* = 0.95. We derived the hypothesized magnitude of effect based on the results of a preliminary pilot study (not reported here). The statistic tool computed that a group of 10–14 participants would have been sufficient in order to find such an effect. To include an adequate number of participants, we thus enrolled 19 participants (11 females, mean age 26.53 years, SD 3.91 years) from the Sapienza University of Rome. They all gave written informed consent prior to participate to the experiment. All participants had normal or corrected-to-normal vision and reported not being color-blind.

### Stimuli

Stimuli were presented in eight locations around the center of a flat screen monitor (LG 16′, 60 Hz refresh rate) placed at about 50 cm from the participant. A black small fixation cross was presented on the center of the screen throughout each trial. Each memory array included eight colored rectangles (each one subtending a region of about 0.4 × 1.7° visual angle) presented at a distance of 4 ± 0.5° from the fixation cross. Each rectangle was either blue or green and had one of four possible orientations (vertical, horizontal, left 45°, and right 45°) randomly chosen. A memory array contained always four blue and four green rectangles. A black bar (of about 0.1° × 3.3°) was presented as a cue, pointing from the center of the screen to one of the eight locations. The cue ends were distant 0.5° from both the center of the screen and the center of the rectangle previously presented in the cued location. All stimuli were presented on a gray background. Stimulus presentation was controlled by E-prime software (version 1.0).

### Procedure

**Figure 1:**
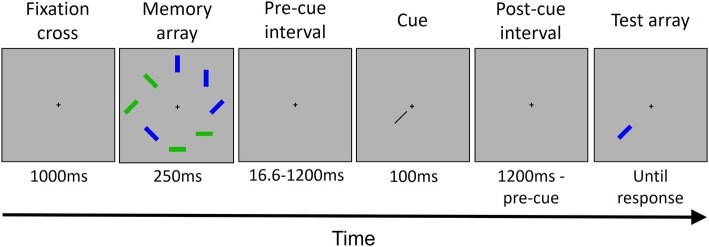
The trial structure. Each trial started with the presentation of a fixation cross, followed by the memory array that included eight colored rectangles. After a variable interval (see Methods Section for details), a cue was presented, pointing to one out of the eight locations occupied previously by one of the rectangles. Then, after another interval, the rectangle in the cued location was presented as a probe. The rectangle remained displayed until the participant gave a response. In this example, the cued rectangle changed its orientation.

Each trial (see Figure 1) started with the presentation of a fixation cross at the center of the screen. Participants were instructed to fixate the cross throughout all the trial. After 1,000 ms, the memory array was presented. The memory array consisted of four blue and four green rectangles randomly placed in eight possible locations around the fixation cross. Each rectangle had an orientation randomly selected out of four possible orientations (vertical, horizontal, and two diagonals). The memory array was presented for 250 ms. After an interval of either 16.6, 200, 600, or 1,200 ms from the offset of the memory array, a cue was presented for 100 ms. The cue was a black bar pointing from the center of the screen toward one of the eight locations previously occupied by a rectangle. The cue indicated a location occupied by a green rectangle in the 80% of trials (high priority color) and a location occupied by a blue rectangle in the 20% of trials (low priority color) for half of the participants, and vice versa for the other half. After the cue offset, another interval was presented. The interval duration depended on the pre-cue interval duration, for a total of 1,200 ms by summing the pre-cue and the post-cue intervals. Then, the rectangle presented in the cued location in the memory array was presented in the same location. In 50% of trials, it has the same orientation that it has in the memory array; in the other 50%, it has a different, new randomly chosen orientation. The rectangle lasted on the screen until participant’s response.

The task of the participant was to indicate if the rectangle has the same or a different orientation, by pressing one of two keys in a keyboard (“a” or “l”). The association between keys and responses was counterbalanced across participants (“a” for “different” and “l” for “same” for even participants, “l” for “different” and “a” for “same” for odd participants). After a response was recorded, a blank screen was presented for 1,000 ms, before a new trial was initiated.

Prior to the proper experimental session, participants performed 48 practice trials, 10 for each of the four possible cue delays for the high priority color condition and 2 for each of the four of possible cue delays for the low priority color condition. After this practice and following verification that the participant had understood the task, each participant completed the 400 experimental trials divided in four blocks of 100 trials each, 20 for each cue delay for the high priority color condition and 5 for each cue delay for the low priority color condition. Each block was separated from the next one by a short rest interval of at least 2 min. The trial sequence for the different conditions was fully randomized within each block. The experiment was administered individually to each subject in a quiet, dark room.

### Experimental Design

In the experimental task, participants were requested to report whether the cued rectangle changed or not the orientation it had in the memory array when presented after the retention interval. A 2 × 4 repeated-measures design was employed with the priority of the cued rectangle color (“high” for the color cued in the 80% of trials; “low” for the color cued in the 20% of trials) and the delay of the cue (16.6, 200, 600, and 1,200 ms) as the two independent variables. The dependent variable was the percentage accuracy in report if the orientation change occurred or not. Moreover, we analyzed with the same experimental design the estimated number of “high” and “low” priority memorized stimuli as dependent variable. See the data screening and analysis section for more details.

### Data Screening and Analysis

Data from trials with reaction times (RTs) shorter than 200 ms or longer than 5,000 ms were removed. This was less than 1.5% of the data. Main analyses reported were conducted by means of within-subjects ANOVA followed by *post hoc t*-tests corrected by Bonferroni’s method for multiple comparisons, in which *p*s were multiplied by the number of comparisons conducted. Moreover, for each effect we reported the partial *η*^2^ as a measure of effect size for the considered effect. We also conducted Bayesian ANOVA by means of JASP software (JASP Team, 2019) in order to confirm the results obtained with the classical inferential statistical methods. This method allows comparing the posterior probability of a null hypothesis (H_0_) with that of a given alternative hypothesis (H_A_) obtained after collecting the data, indicating which one of the two are most likely to be true based on the observed evidence. Thus, it allows to directly support one of two alternatives hypotheses, not only to reject the null hypothesis as for the classic inferential methods, as well as to have an indication of the contribution of each variables in incrementing the model fitting to the data. These advantages strongly support the use of the Bayesian methods for hypotheses testing (see Wagenmakers et al., 2018). For each analysis, we reported the Bayes factors along with their interpretation for each model including main effects or their interaction. Moreover, we reported also the pairwise comparisons of interest conducted with the same method.

## Results

**Figure 2:**
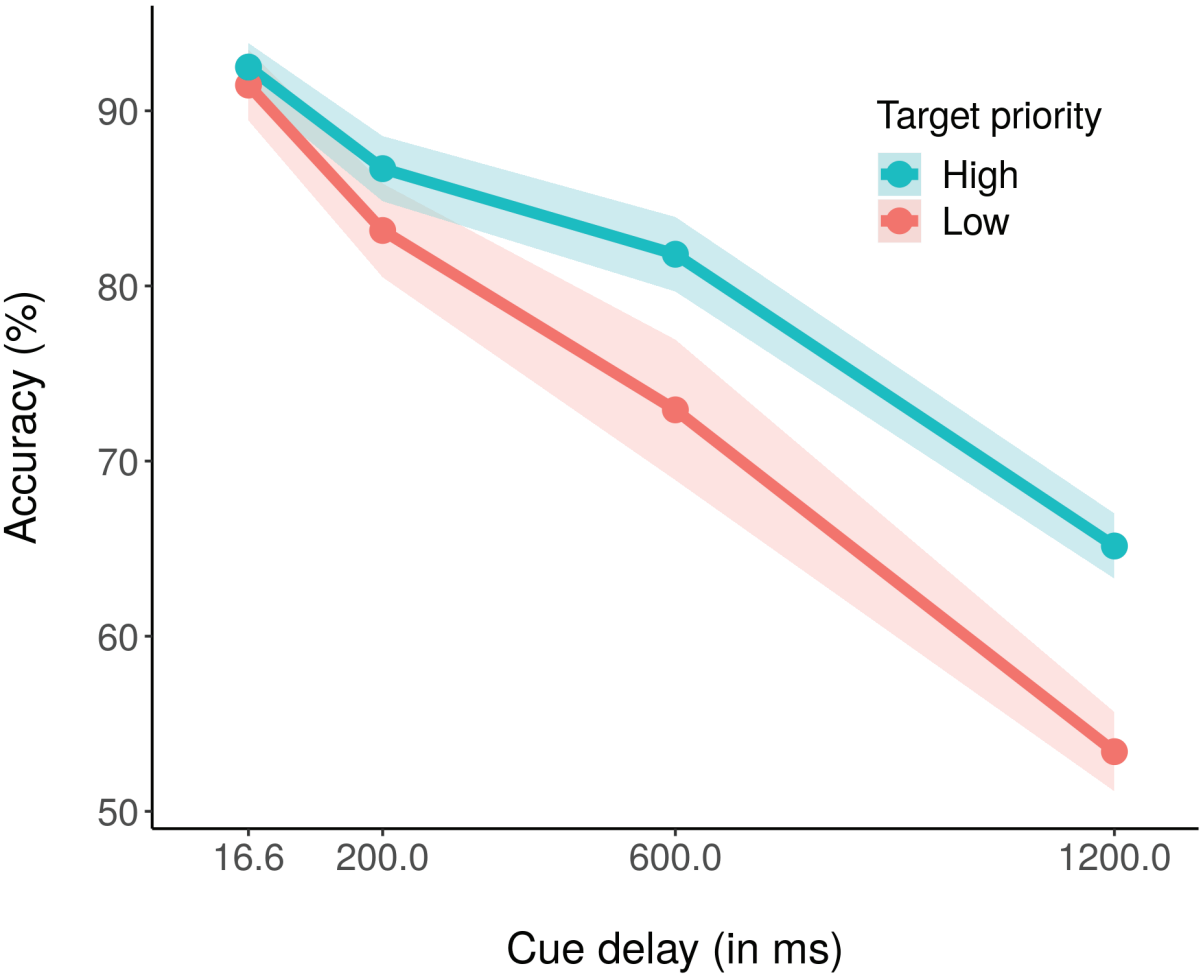
Mean percentage accuracy in the high (blue coded) and the low (red coded) priority conditions for the four cue delays. The shaded area around each line stands for the standard error of the means.

The average percentage of correct report accuracy across conditions was 78.39% (chance level = 50%). Figure 2 shows the average percentage of correct report accuracy as a function of cue delay and color priority. A within-subjects ANOVA with priority (high, low) and cue delay (16.6, 200, 600, and 1,200 ms) as independent variables and average accuracy as dependent variable revealed significant main effects of both priority, *F*(1,18) = 16.78, *p* < 0.001*, partial η*^2^ = 0.48, and cue delay, *F*(3,54) = 118.30, *p* < 0.001, *partial η*^2^ = 0.87. For the main effect of priority, it was evident that accuracy was higher for the high priority color (81.51%) as compared to the low priority color (75.26%). For the main effect of cue delay, performance decreased as the delay increased, with an accuracy of 91.99% for 16.6 ms of delay, 84.91% for 200 ms of delay, 77.41% for 600 ms of delay, and 59.25% for 1,200 ms of delay. The ANOVA also revealed a significant priority × cue delay interaction effect (Figure 2), *F*(3,54) = 3.76, *p* < 0.05*, partial η*^2^ = 0.17. *Post hoc* corrected *t*-test analyses revealed that while accuracy did not differ between high- and low-priority stimuli with 16.6 or 200 ms of cue delay, respectively, *p* = 0.32 and *p* = 0.16, accuracy for low priority stimuli was significantly lower than accuracy for high priority stimuli with both 600 ms, *t*(18) = −2.52, *p* < 0.05, and 1,200 ms of cue delay, *t*(18) = −3.88, *p* < 0.01. We also conducted pairwise corrected *t*-tests within each priority condition, to assess if and at which delay the performance started to decrease. For the high priority condition, performance did not significantly decrease between cue delay 16.6 (92.50%) and 200 ms (86.70%), *p* = 0.16, nor between cue delay 200 and 600 ms (81.80%), *p* = 0.37, but it significantly decreased between cue delay 600 and 1,200 ms (65.08%), *t*(18) = 7.31, *p* < 0.01. Instead, for the low priority condition, performance did not decrease only between cue delay 16.6 (91.49%) and 200 ms (83.11%), *p* = 0.26, whereas it significantly decreased between cue delay 200 and 600 ms (73.02%), *t*(18) = 3.24*, p* < 0.05, and between cue delays 600 and 1,200 ms (53.42%), *t*(18) = 5.53, *p* < 0.01.

As a further test for the difference between high and low priority performance under different conditions, we conducted a paired *t*-test by comparing performances at the iconic memory time delay (16.6 ms) and at the fragile VSTM time delay (1,200 ms) in high and low priority conditions. The test revealed that the performance difference between 16.6 and 1,200 ms was significantly lower for the high priority condition (27.35%) than for the low priority condition (38.06%), *t*(18) = −3.70, *p* < 0.1. This result showed a significantly slower decrease of performance with cueing time in the high priority as compared to the low priority condition.

To confirm this pattern of results, we also conducted a Bayesian ANOVA with the two factors of target priority and cue delay, along with their interactions. Table 1 reports the results of such analysis. As shown, compare to the null model, all other models received strong support from the data (BF_10_ > 100) except for the model with only the target priority factor, that was weakly supported (BF_10_ = 2.46). The most supported model was the model including the two main factors but not the interaction term; in fact, the analysis supported that this was 1.54 times more likely. However, adding the interaction term to the model increased the Bayes factor of 2.52, suggesting that including such term increased the posterior model odds. This pattern of results confirmed the results obtained with the classical ANOVA reported before, indicating that both our main factors as well as their interaction should be included to obtain a good model of the data. *Post hoc* Bayes t-test comparisons between the two target priorities for each cue delay showed no support for a difference with cue delay 16.6 ms, BF_10_ = 0.34, and 200 ms, BF_10_ = 0.50, while a weak support, BF_10_ = 1.37, and a strong support, BF_10_ = 86.50, for a difference between high and low priority condition with cue delay 600 and 1,200 ms, respectively. For the high priority condition only, post-hoc comparisons testing the difference in accuracy between consecutive cue delays showed a moderate support for a difference between 16.6 and 200 ms cue delay, BF_10_ = 3.41, while no support with 200 and 600 ms cue delay, BF_10_ = 1.00, and a very strong support with 600 and 1,200 ms cue delay, BF_10_ > 150. We found very similar results while considering only the low priority condition, with the only dissimilarity of a weak support to a difference in accuracy between cue delay 200 and 600 ms, BF_10_ = 1.78. To sum up, the Bayesian *post hoc* analysis confirmed that the difference in performance between the two priority conditions were supported only with long cue delays and that performance dropped from 200 to 600 ms cue delay only in the low priority condition.

**Table 1:** Model comparison for the Bayesian ANOVA on accuracy.

Models	P(M)	P(M|data)	BF_M_	BF_10_	Error%
Null model	0.20	1.00e−25	4.03e−25	1.00	—
Cue delay	0.20	0.01	0.03	8.57e+22	0.01
Target priority	0.20	2.47e−25	9.89e−25	2.46	1.01e−7
Target priority + cue delay	0.20	0.60	6.12	6.01e+24	1.40
Target priority + cue delay + target priority × cue delay	0.20	0.39	2.52	3.84e+24	2.64

**Figure 3:**
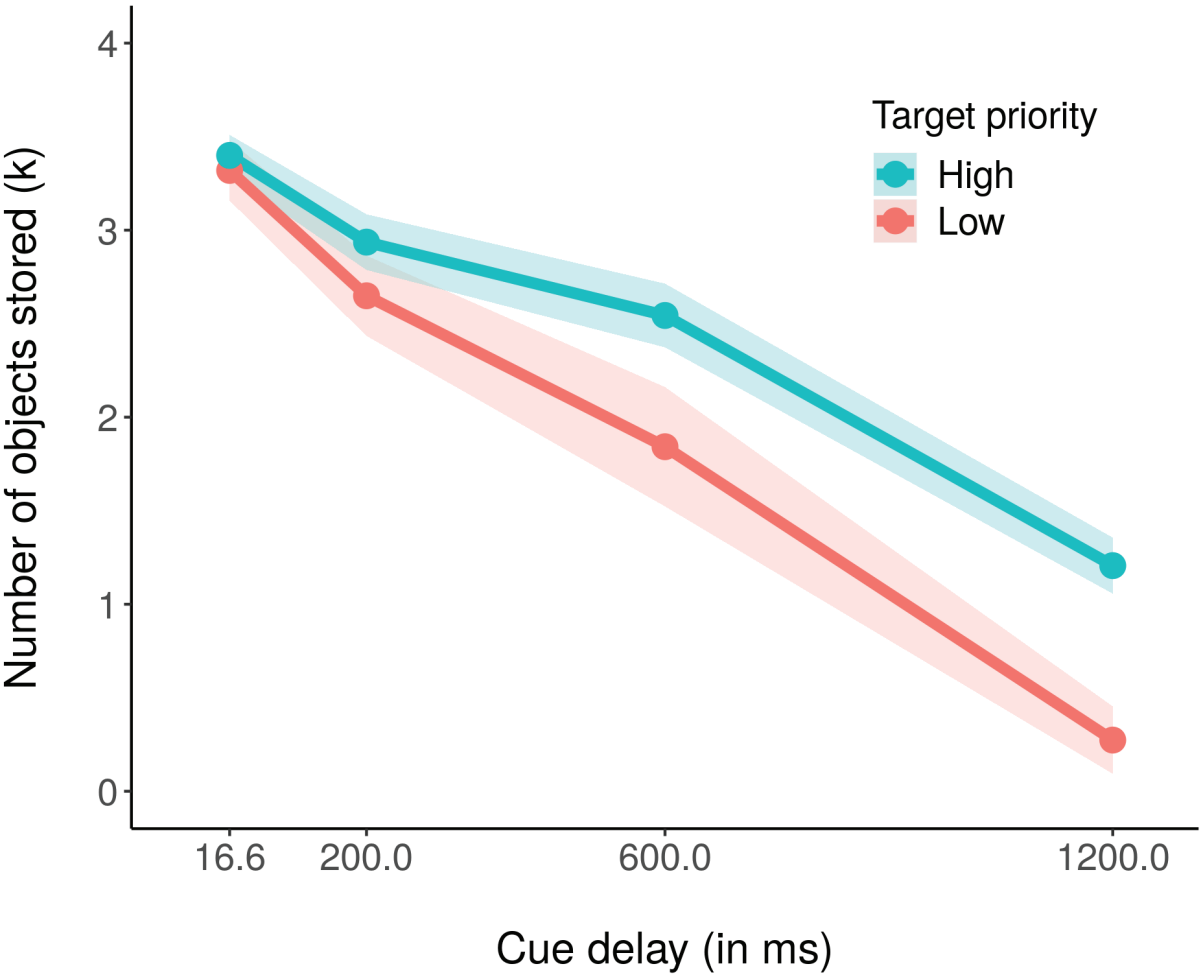
Mean number of estimated stored objects in visual working memory (*k*) in the high (blue coded) and low (red coded) priority conditions for the four cue delays. The shaded area around each line stands for the standard error of the means.

We subsequently computed the estimated number of objects stored in visual working memory, in terms of the *k* index (Cowan, 2001; Rouder et al., 2011). The formula for computing the *k* is the following: *k* = *N* (*H* − *F*); where *k* is the estimated number of stored objects, *N* is the set size (number of objects in the memory array), *H* is the hit rate (when the participant responds “change” and the probed object actually changed), and *F* is the false alarm rate (the participant responds “change,” but the probed object did not actually change). In particular, we computed the *k* for each priority condition in all the cue delay conditions (see Figure 3). We found that participants encoded on average 2.52 out of the four high-priority stimuli, but only 2.02 out of the four low priority ones. Moreover, the total number of stored objects decayed with the cueing time, starting with 6.72 objects encoded when the cue was presented immediately after the memory array, 5.58 objects at 200 ms, 4.38 objects at 600 ms, and finally with 1.48 objects stored with the longest delay of 1,200 ms. These results were confirmed by a 2 (priorities) × 4 (cue delays) within ANOVA on average *k*, that revealed a significant main effect of both priority, *F*(1,18) = 16.47, *p* < 0.001, partial *η*^2^ = 0.48, and cue delay, *F*(3,54) = 119.74, *p* < 0.001, partial *η*^2^ = 0.87. We also found a significant interaction effect, *F*(3,54) = 3.67, *p* <0.05, partial *η*^2^ = 0.17, with more high priority objects stored as compared to low priority ones when the cue delay was 600 ms (*k*_high priority_ = 2.54 and *k*_low priority_ = 1.84, *t*(18) = −2.47, *p* < 0.05) and 1,200 ms (*k*_high priority_ = 1.21 and *k*_low priority_ = 0.27, *t*(18) = −3.84, *p* < 0.01). As for the accuracy effect, the Bonferroni corrected pairwise *t*-tests within each priority condition revealed that the amount of stored objects decreased significantly in the high priority condition only between cue delay 600 (*k* = 2.54) and 1,200 ms (*k* = 1.21), *t*(18) = 7.30, *p* < 0.01, whereas for the low priority condition, it significantly decreased in all pairs of consecutive cue delays 200 (*k* = 2.65) to 600 ms (*k* = 1.84), *t*(18) = 2.80, *p* < 0.01, and from 600 and 1,200 ms (*k* = 0.27), *t*(18) = 5.62, *p* < 0.01.

Also for the *k*, we conducted a Bayesian ANOVA including target priority, cue delay, and their interactions as independent variables. Table 2 reports the results of this analysis. We obtained strong support for all the models as compared to the null model (BF_10_ > 100) with the exception of the target priority model, that received only weak support from the data (BF_10_ = 2.38). As for the accuracy, the best model was that with both the independent variables but without the interaction term, but adding this increased the Bayes factor of 2.44. These results argued again in favor of a model including both the independent variables as well as their interaction, confirming the results obtained with the ANOVA, in which all the investigated factors was significantly related to the data. The *post hoc* Bayes *t*-test comparisons showed a comparable pattern of results of that reported above for the accuracy.

**Table 2:** Model comparison for the Bayesian ANOVA on *k*.

Models	P(M)	P(M|data)	BF_M_	BF_10_	Error%
Null model	0.20	8.14e−26	3.25e−25	1.00	–
Cue delay	0.20	0.01	0.04	1.12e+23	0.01
Target priority	0.20	1.94e−25	7.34e−25	2.38	1.09e−7
Target priority + cue delay	0.20	0.60	6.30	7.52e+24	1.95
Target priority + cue delay + target priority × cue delay	0.20	0.38	2.44	4.66e+24	2.21

## Discussion

In our study, we originally addressed the interaction of visual attention (priority-related attentional bias) with different stages of visual processing and storage, with relevance to characterize the role of attention at different stages of phenomenal consciousness, before access consciousness. To this aim, we used a change detection procedure linked to a visual working memory task with cueing of the object location for access consciousness and report with different delays after offset of the memory array. The delays were chosen to match different stages of visual processing and storage from iconic memory (with two intervals) to an intermediate fragile visual short-term memory (with two intervals), with subsequent encoding (storage) in and retrieval from visual working memory. We introduced a different priority (high vs. low) of the objects in the memory array in order to investigate the interaction of priority with the cueing delay, i.e., the effect of the attentional bias at different stages of visual processing and maintenance before cueing the target location for access in visual working memory.

In line with our hypothesis, we found that both in terms of percentage accuracy and estimated storage capacity *k* the visual attention bias was effective at the longer cueing delays (600 and 1,200 ms), which are plausibly related to a fragile short-term memory stage, but not at the shorter cueing delays (16.6 and 200 ms), which are plausibly related to a preattentive iconic memory stage. Given that both iconic memory and the fragile short-term memory have been linked to phenomenal consciousness (Lamme, 2003, 2006; Block, 2007, 2011; Sligte et al., 2008), our findings suggest two stages of phenomenal consciousness, or broad cognitive accessibility, the first of which preattentive (in line with Block, 2007), and the second modulated by visual attention. The latter component in particular appears original in light of the relevant literature on both conscious access and phenomenal consciousness (e.g., Baars, 1997; Dehaene et al., 2006; Block, 2007; Dehaene and Changeux, 2011), given that attention has been selectively associated to access consciousness, while phenomenal consciousness, or a preconscious state of representation, to a preattentive stage.

Our results thus suggest two subsets of information, a preattentive set I2A and an attention-modulated set I2B, in between unconscious information and conscious access, with reference to the taxonomy of Dehaene and Naccache (2001) considered above. Thus, the present findings highlight that phenomenally conscious and access conscious representations may not only share recurrent neural processing in the brain but also the influence of attention, after the stage of preattentive iconic memory representation. The study also originally suggests that access consciousness or narrow cognitive accessibility, in terms of probability to transfer a target at a probed location in visual working memory after a given delay, depends upon the attentional modulation of object representations for broad cognitive accessibility. Thus, the latter should be conceived as not just a passive buffering of supraliminal stimuli waiting for a sudden attentional amplification when the stage of conscious access begins (Dehaene et al., 2006).

Our findings reveal a clear increase of the biased competition between high and low priority objects over time after offset of the memory array, which can be linked to a gradual amplification of the neural representation of high-priority objects and a gradual suppression of the neural representation of low-priority objects. Such neurodynamics of amplification and suppression related to phenomenal consciousness and broad cognitive accessibility (set I2B) appear gradual rather than all-or-none or ignition-based as for the stage of conscious access in the workspace (see Dehaene et al., 2003, for the latter). Our findings appear thus also relevant in light of the debate about the gradual versus all-or-none nature of conscious representations (Dehaene et al., 2003; Overgaard et al., 2006; Raffone and Pantani, 2010; Windey and Cleeremans, 2015).

Our results also highlight that the state of the fragile visual short-term memory changes over time with an increasing influence of visual attention. This process is plausibly mediated by evolving recurrent excitatory and inhibitory interactions between multiple areas of visual cortex. By the definition itself of phenomenal consciousness, such changes of the involved representations can be assumed to be linked with changes in the subjective (iconic) experience of the memory array over time with an increased prominence of the high-priority objects versus the low-priority objects, besides the bias for broad accessibility for high- versus low-priority objects. Thus, the present evidence suggests that the fragile (intermediate) visual short-term memory store between iconic and visual working memory is sensitive to attentional bias in a time-dependent manner, with a stable state (attractor) reflecting the attentional bias reached between 600 and 1,200 ms. Remarkably, phenomenal consciousness would not be stationary but would rather be dynamic due to intrinsic dynamics and attention, before conscious access.

**Figure 4:**
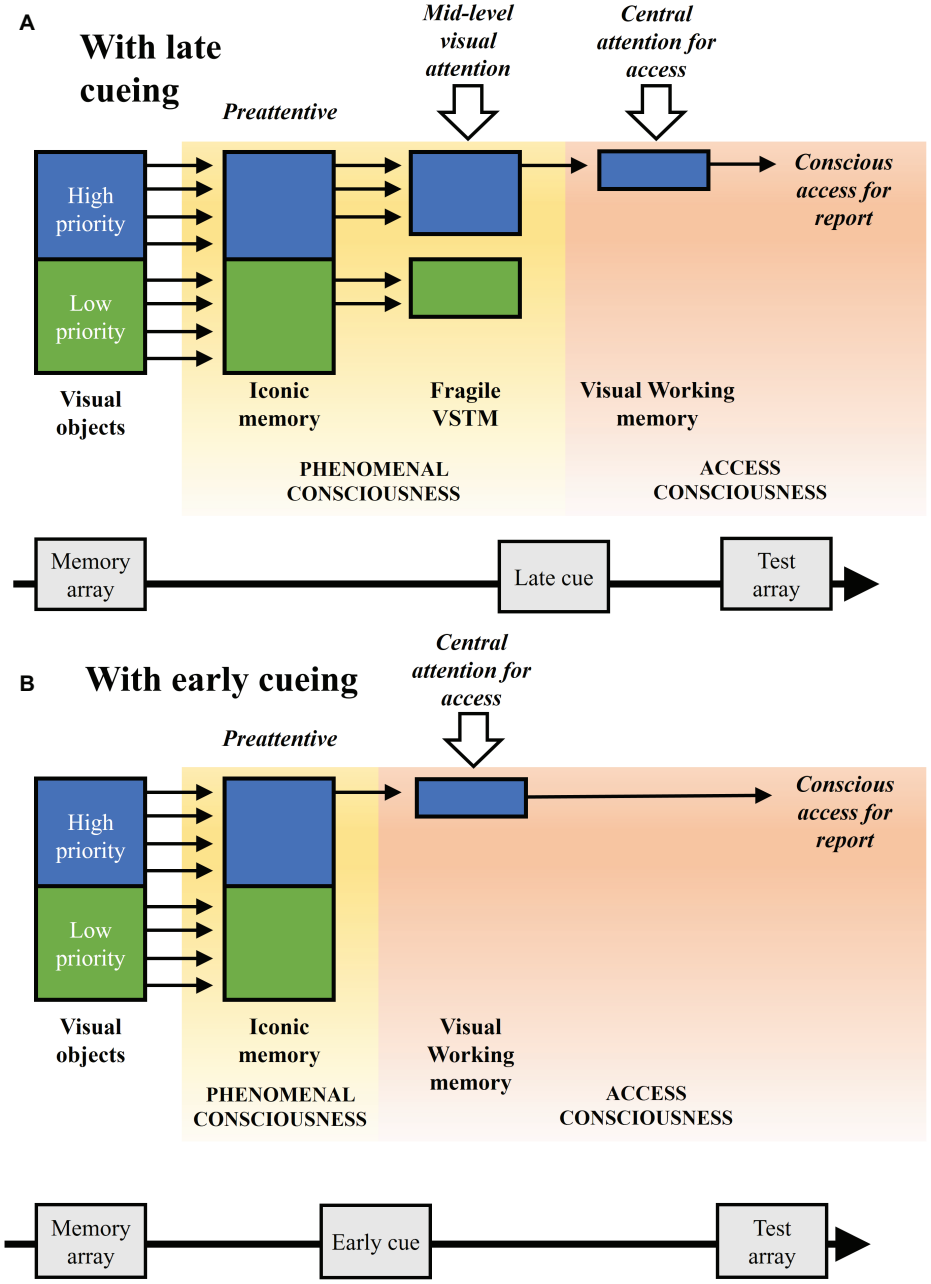
Illustration of the visual processing and maintenance stages associated to the experimental paradigm, with their related attention and consciousness components, considering the theoretical implications of the study and other supporting empirical and theoretical research. **(A)** The implicated stages with late cueing. Low- and high-priority visual objects are presented and then preattentively represented in iconic memory, as a first component of phenomenal consciousness. At a subsequent fragile visual short-term memory (VSTM) stage object representations are biased in a time-dependent manner by a mid-level visual attention based on their priority, as a second component of phenomenal consciousness. When the cue appears, access consciousness takes place directed by a central attention, as related to encoding in visual working memory. Access consciousness further operates for report, as related to retrieval from visual working memory. **(B)** The direct transition from iconic memory to the stage of conscious access with early cueing, without the intermediate stage of the fragile VSTM and with a longer visual working memory maintenance, thus with a shorter stage of phenomenal consciousness and a longer stage of access consciousness as compared to the condition with late cueing illustrated in **(A)**.

In our experimental paradigm, visual attention is driven in a top-down manner by priorities associated to the color of the objects, which are however not response relevant *per se*. Such visual attention affects the stages of visual processing and maintenance before conscious access, but not the latter stage, which is driven by the cue at the object location relevant for the change detection report. We can refer to the latter form as attentional selection for conscious access (see also Raffone and Pantani, 2010). This proposal relates to the suggested distinction between a *central attention*, with a serial nature, and the processes of *mid-level* and *peripheral attention*, with a parallel nature and implicating distinct representational systems which can be maintained simultaneously with little or no interference (Tamber-Rosenau and Marois, 2016). However, a mid-level visual attention, as plausibly involved in the present study, might also filter objects serially with rapid shifts, while the mechanism of central selection for access would operate serially on a slower time scale (Raffone et al., 2014). Figure 4 illustrates the different stages of visual processing and maintenance, as well as attentional and consciousness components, linked to our experimental paradigm, and highlighting the theoretical implications of our findings, also on the basis of other empirical findings and theoretical insights reviewed in this article.

We overall found a lower maintenance before cueing as compared to the earlier study of Landman et al. (2003), which can be explained by a reduced grouping (chunking) of the objects in the memory array using two colors. The different priorities of the objects might have further concurred to prevent chunking effects based on orientation similarity. In neurophysiological and neurocomputational terms, chunking of neural representations with different firing rates (related to different priorities) might have been more difficult, such as for a synchrony-based mechanism (e.g., Fries et al., 2001; Raffone and Wolters, 2001).

Further experiments are needed to clarify the involved processes and mechanisms, including event-related potentials (ERPs) with the experimental procedure used in this study. In ERPs research, two main correlates of consciousness have been proposed, an early one (N200, visual awareness negativity), which has been related to phenomenal awareness, and a later one (P3), which has been linked with access consciousness (Salti et al., 2012; Koivisto et al., 2016, 2017; Koivisto and Grassini, 2016). These correlates also support different theories on the fast emergence of consciousness or the global workspace theory. We can hypothesize a modulation of visual awareness negativity by visual attention, e.g. by manipulating the priority of visual objects in the right and left hemifield, as also related to phenomenal consciousness. A location-based priority bias could also be usefully investigated as related to a contralateral delay activity (Vogel and Machizawa, 2004), as a function of the cueing delay. The effect of bottom-up attention, such as bottom-up attention driven by sudden onsets (Woodman et al., 2003), can also be further investigated in terms of attentional bias with different cueing delays. Also, the bias related to emotional stimuli, such as schematic faces displaying different emotions (Simione et al., 2014), as a function of the cueing delay, can be usefully investigated with the paradigm used in this study. We hypothesize the involvement of similar mechanisms of attention-related bias for phenomenal consciousness or broad cognitive accessibility, with a possible earlier effect of bottom-up attention at the stage of iconic memory, i.e., 200 ms after offset of the memory display. Moreover, the effects of top-down and bottom-up attention can be contrasted in the same paradigm, with assessment of the resulting attentional bias with different cueing delays. Finally, dedicated neurocomputational investigations with an explicit simulation of the experimental setting used in this experiment can clarify the involved neural mechanisms and lead to new predictions to be tested in further experiments.

We also observe the need to verify if preattentive iconic memory (information set I2A) also depends on some form of attention (namely spatial attention) given the findings of Koivisto et al. (2009) and, Koivisto and Revonsuo, 2010 showing the dependence of visual awareness on spatial attention, and those of Donovan et al. (2017) suggesting that spatial attention is a necessary precursor for object-based attention. Possibly a cascade of attentional processes, from peripheral to mid-level to central attention, modulate different stages of phenomenal consciousness or direct conscious access, i.e., the information sets I2A, I2B, and I3. Further investigations are necessary to clarify such attentional effects and their interplay with different facets of consciousness, e.g., with variations on the experimental paradigm used in the present work.

To conclude, the present study shows that a simple behavioral paradigm can shed light on stages of visual processing and maintenance related to two components of phenomenal consciousness and their interplay with visual attention. Variations on our experimental paradigm can investigate the effects of different attentional processes and thus enable the study of their influences on multiple stages of visual processing and maintenance, and on the related consciousness components. The study also remarkably highlights a dissociation between a mid-level visual attention modulating phenomenal consciousness and a central attention directing access consciousness.

## Ethics Statement

The study was approved by the Research Ethics Committee at the Sapienza University of Rome and was carried out in line with the ethics guidelines of the National Board of Italian Psychologists and with the 1964 Helsinki declaration and its later amendments or comparable ethical standards. Informed consent statement was obtained from all participants before starting the experimental session.

## Author Contributions

LS, EP, AR, and SC discussed the original idea of the project. LS programmed the experimental software. EP and SC conducted the experiment and collected the data. LS and SC conducted the data analysis. LS and AR wrote the first draft of the paper. EP and SC reviewed the draft.

### Conflict of Interest

The authors declare that the research was conducted in the absence of any commercial or financial relationships that could be construed as a potential conflict of interest.
